# Non-contact imaging of peripheral hemodynamics during cognitive and psychological stressors

**DOI:** 10.1038/s41598-020-67647-6

**Published:** 2020-07-02

**Authors:** Daniel McDuff, Izumi Nishidate, Kazuya Nakano, Hideaki Haneishi, Yuta Aoki, Chihiro Tanabe, Kyuichi Niizeki, Yoshihisa Aizu

**Affiliations:** 1 Microsoft Research, Redmond, USA; 2 Tokyo University of Agriculture and Technology, Tokyo, Japan; 3 Chiba University, Chiba, Japan; 4 Yamagata University, Yamagata, Japan; 5 Muroran Institute of Technology, Hokkaido, Japan

**Keywords:** Biomedical engineering, Human behaviour

## Abstract

Peripheral hemodynamics, measured via the blood volume pulse and vasomotion, provide a valuable way of monitoring physiological state. Camera imaging-based systems can be used to measure these peripheral signals without contact with the body, at distances of multiple meters. While researchers have paid attention to non-contact imaging photoplethysmography, the study of peripheral hemodynamics and the effect of autonomic nervous system activity on these signals has received less attention. Using a method, based on a tissue-like model of the skin, we extract melanin $\text {C}_{m}$ and hemoglobin $\text {C}_{HbO}$ concentrations from videos of the hand and face and show that significant decreases in peripheral pulse signal power (by 36% ± 29%) and vasomotion signal power (by 50% ± 26%) occur during periods of cognitive and psychological stress. Via three experiments we show that similar results are achieved across different stimuli and regions of skin (face and hand). While changes in peripheral pulse and vasomotion power were significant the changes in pulse rate variability were less consistent across subjects and tasks.

## Introduction

Quantitative evaluation of peripheral hemodynamics provides a valuable way of monitoring tissue metabolism, physiological state and health. Peripheral hemodynamics can be characterised via the plethysmograph and vasomotion signals. These capture changes in blood volume in the surface of the skin and spontaneous oscillations in tone of blood vessel walls, independent of heart beat, innervation or respiration. Contact sensors have traditionally been the only way to capture measurements of the plethysmogram and vasomotion. However, recent advances in imaging technology and computational methods have enabled non-contact imaging-based measurement of these signals. Imagers have several advantages over contact devices. First, they remove the need for uncomfortable sensors that may in and of themselves impact someone’s physiological state. Second, they allow for spatial measurement and visualization of the peripheral signals using only one sensor.

**Figure 1:**
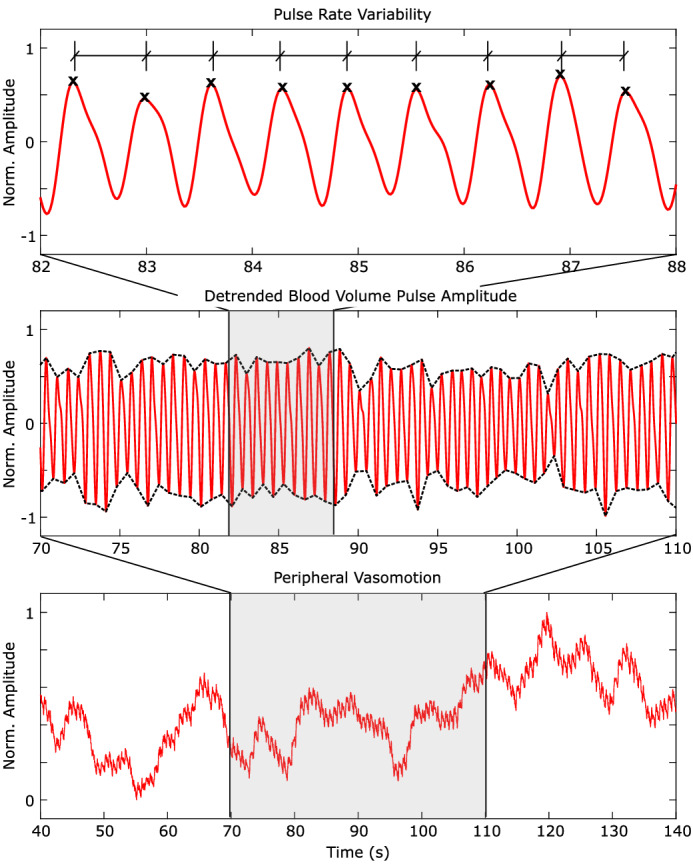
Peripheral hemodynamics contain rich information about physiological state and health. We present analysis of non-contact camera-based measurements of pulse rate variability, blood volume pulse amplitude and vasomotion signals during three experiments eliciting stress.

In this paper, we investigate how cognitive and psychological stress impacts imaging-based measures of pulse rate variability, the peripheral pulse amplitude and vasomotion. Figure 1 illustrates these signals at different time scales. The plethysmogram is the periodic variation in blood volume due to the cardiac pulse traveling through the body. Photoplethysmography (PPG), is an optical approach for measuring the blood volume pulse (BVP) signal and has been widely used to evaluate cardio-vascular functions such as heart rate, blood pressure, cardiac output, vascular compliance^1^. The peripheral blood volume pulse waveform envelope pinches when a person is startled, fearful or anxious, which is the result of the body diverting blood from the extremities to the vital organs and working muscles, to prepare them for action, the “fight or flight” response. Use of this phenomenon in affective computing applications is well established and has been leveraged to capture emotional responses in marketing/media testing^2^, computer tasks^3^, in training machine learning systems^4^ and many psychological studies^5, 6^.

Vasomotion is the spontaneous low-frequency oscillation in the diameter of blood vessels with 1–4 cycles/min ($\alpha$ waves) and 4–8 cycles/min ($\beta$ waves), independent of the cardiac pulse or respiration. Vasomotion has been associated with the reduction of vascular resistance, prevention and reduction of edema, regulation of body or skin temperature, and cancellation of hypoxic regions in capillary plexuses^7^. Both the photoplethysmogram and vasomotion have been linked to autonomic nervous system (ANS) activity. However, relative to understanding of the plethysmogram, the role of vasomotion and the mechanisms by which it occurs and is controlled are still somewhat unclear^8^. Vasomotion has been linked to health conditions, including diabetes^9^ and we will argue that it is a signal that has been under utilized with respect to measuring the body’s response to stressful stimuli. If ubiquitous imaging devices can be used to measure vasomotion reliably, then computer vision could be a very useful tool in tracking changes in ANS activity.

Fluctuations in the intervals between subsequent heart beats, known as interbeat intervals (IBI), capture heart rate variability (HRV) or when measured from the photoplethysmogram, pulse rate variability (PRV). HRV and PRV are similar physiological measures and are both influenced by autonomic nervous system activity. The ratio between low frequency power, in the range 0.04 Hz to 0.15 Hz, and high frequency power, in the range 0.15 Hz and 0.4 Hz, typically increases with sympathetic nervous system activity. PRV has been given considerably more attention when compared to peripheral pulse amplitude and vasomotion.

Non-contact video-based physiological measurement is a fast growing research domain as it offers the potential for unobtrusive, concomitant measurement and visualization of important vital signs using ubiquitous sensors (e.g., low-cost webcams or smartphone cameras)^10^. Non-contact measurement of physiological signals has some distinct advantages in certain applications. In Neonatal Intensive Care Units (NICUs) the monitoring of vital signs is essential; however, infants are vulnerable. Attaching electrodes to their bodies (the current standard of care) can lead to skin damage, that could increase the chance of infections, or discomfort to the baby. Patients with severely damaged skin, for example burn victims, also experience discomfort from contact devices. Beyond healthcare, non-contact measurement offers applications and benefits in ubiquitous computing applications, unobtrusive measurement of physiological responses in psychological studies and long-term tracking^11^.

Early efforts in imaging found that spatial aggregation of pixels boosted the signal-to-noise ratio sufficiently to recover the reflectance photoplethysmogram^12^. More robust methods for recovering the BVP signal have been subsequently proposed^13–17^. These have enabled the measurement of pulse rate variability^14, 18^ and respiration signals via respiratory sinus arrhythmia^14^. Non-contact sensing of psychological stress could have many application. In high-stress work environments (e.g., air traffic or military control centers) understanding the stress levels of personnel could help increase safety. In learning contexts measures of cognitive stress or load could help with the design of intelligent tuition systems that automatically adapt to a student's current state. Using imaging photoplehysmography researchers have used PRV to measure autonomic nervous system activity correlated with cognitive load^19–21^. PRV measurement is challenging due to the requirement to detect inter-beat intervals accurately. Other methods of measuring autonomic nervous system activity via non-contact imaging devices primarily used thermal cameras^22^. These devices are not ubiquitous and are costly relative to digital RGB cameras. We argue, therefore, that more efforts should be invested into gathering complementary signals that capture changes in autonomic nervous system activity. In this work, we will focus on the use of RGB imagers in recovering changes in the photoplethysmogram and vasomotion signals during stress inducing tasks.

Most iPPG methods evaluate the plethysmogram based on the periodic changes in the red, green, and/or blue color channels, without the quantification of hemoglobin content. Furthermore, in these methods, only the plethysmogram is selectively highlighted and the fluctuation due to vasomotion are removed from the raw signals via detrending and/or bandpass filtering. An alternative, non-contact imaging photoplethysmography method, based on a tissue-like model of the skin, allows accurate recovery of the BVP, average heart rate (HR) and pulse rate variability^23, 24^. Most significantly, this method allows measurement and visualization the contents of melanin $\text {C}_{m}$, oxygenated hemoglobin $\text {C}_{HbO}$, and deoxygenated hemoglobin $\text {C}_{HbR}$ in human skin. Furthermore, this approach does not require detrending, normalization or other signal processing steps that remove low-frequency oscillations or transform the amplitude of the pulse signal. Thus we can extract pulse amplitude and vasomotion information directly.

In this paper, we present: (1) experiments using three different stimuli to elicit cognitive and psychological stress, (2) analyses and meta-analyses of remotely measured imaging-based measures of the plethysmogram, vasomotion and pulse rate variability signals, (3) visualizations the contents of blood in these videos. We find significant decreases in peripheral pulse amplitude and vasomotion during periods of stress. We also find that pulse rate variability measures have significantly higher individual variability and the differences were not significant under stress versus not.

## Related work

### Imaging photoplethysmography

The principle of measuring peripheral blood flow via imaging devices^12, 25^ has received increasing attention. Approaches that are more amenable to computation and that leverage advances in computer vision have increased how robust these methods are^13, 15, 17, 18^. Initially iPPG was used to estimate heart rate. Subsequently, focus has extended to pulse rate variability and respiratory sinus arrhythmia^13, 14^, blood oxygenation^26, 27^ and pulse transit time^28^.

Informing, or grounding, the decomposition of color channel signals in physically based models has been shown to provide better results than relying on unsupervised decomposition (e.g., ICA or PCA)^15, 24^. Adding additional information in the form of supervised learning, in which a model is trained on a labeled set of data, and then deployed on a separate set also improves the signal-to-noise ratio (SNR) of the BVP and the accuracy of the HR estimates^17^.

Most iPPG algorithms involve a form of unsupervised or supervised learning in order to recover the blood volume pulse. These computational steps are problematic if the aim is to recover vasomotion and pulse amplitude measurements. First, almost all signal processing approaches involve a normalization (e.g., Z scoring) or detrending (e.g., using a smoothness priors approach^29^) step that removes important low-frequency information that is needed for calculating vasomotion. Second, windowed blind source separation techniques such as Independent Component Analysis (ICA) do not preserve amplitude consistently from window to window and thus make it difficult to recover absolute measures of peripheral pulse amplitude or even relative changes if the windows are small. Third, most computational methods do not have a rigorous physiological basis for recovering hemoglobin, melanin and deoxyhemoglobin estimates, reducing their explanatory power.

In existing literature a method was proposed for the measurement of blood concentration in perhipheral tissue via a physical model of the skin tissue^23, 30^. The measurement of human skin hemodynamics was then demonstrated using this method. In this approach the RGB-values are converted into the tristimulus values in the CIEXYZ color space. This step provides a device independent color representation. A Monte Carlo simulation (MCS) of light transport in a human skin model is conducted to empirically identify the relationship between the tristimulus XYZ-values and the concentrations of oxygenated hemoglobin $\text {C}_{HbO}$ and deoxygenated hemoglobin $\text {C}_{HbR}$. The total hemoglobin concentration $\text {C}_{HbT}$ can then be calculated simply as a sum of $\text {C}_{HbO}$ and $\text {C}_{HbR}$. We leverage this approach in our work as it allows us to accuractly recover the signal amplitudes. For a more in-depth survey of iPPG techniques see^10, 31^.

### Imaging measurements of stress

The heart is modulated by the parasympathetic and sympathetic branches of the autonomic nervous system (ANS). Measures of heart and pulse rate variability capture aspects of these changes. Those under mental stress showed reduced HF HRV components compared to a control group^32^. During an attention task lower total HRV power was observed, in addition to a change in heart rate, when compared to baseline^33^.

The first attempts to measure ANS activity using RGB imaging techniques focused on similar PRV statistics^19, 20^. Both time domain PRV statistics^34^ and frequency domain statistics^20^ have been studied. However, waveform amplitude has also been employed^34^. Applying the first and second derivatives to PPG waveforms is useful for determining stress levels^35^ and these waveform morphological signals can be measured using imaging-based techniques^36^. One method^37^ proposed a novel photoplethysmogram-based stress induced vascular index (sVRI) to measure cognitive load and stress. However, to our knowledge this is the first work to look at the effects of stress on the peripheral pulse amplitude and vasommotion measured remotely across different tasks.

### Visualizing peripheral hemodynamics

Imagers have the distinct advantage over traditional PPG sensors in that the signals can be visualized spatially and in an intuitive fashion. There are several approaches that can be used to magnify subtle signals with videos. Lagrangian techniques^38^ were early examples; however, while these are suitable for amplifying motions, they are not good at amplifying color changes. Eulerian video magnification (EVM)^39^ on the other hand, is a magnification technique that can be used for color changes. EVM does not preserve the waveform morpohlogy of the pulse signal perfectly. Subsequent work proposed the use of phase-based features^40^. Several approaches have improved the robustness to head motions using supervised learning^41, 42^.

## Results

**Figure 2:**
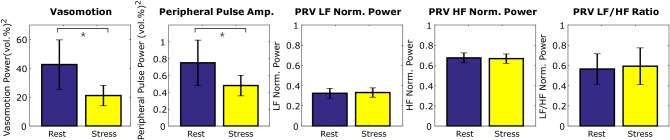
(i) Vasomotion, (ii) peripheral pulse amplitude, (iii) PRV LF normalized power, (iv) PRV HF normalized power and (v) PRV LF/HF power ratio across all three tasks for the rest and stress conditions. The error bars reflect the standard errors.

**Figure 3:**
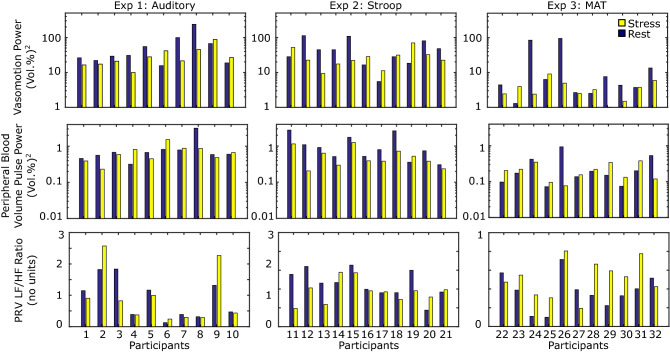
Individual-level measures of: (top row) vasomotion power, (middle row) peripheral blood volume pulse power, (bottom row) PRV LF/HF ratio. For each individual we calculated a single measure per condition.

First, the total blood concentration ($\text {C}_{tb}$) signal was extracted from each video (see Fig. 7). Then we compared the pulse rate variability LF/HF ratio, integral power of the photoplethysmogram and vasomotion signals. We compared each signal for the period before the onset of the stimulus with the signal during the stimulus period. Figure 2 shows the average results. The PRV LF/HF ratio is dimensionless and the power of the PPG and vasomotion signals is measured in vol(%)$^2$. Figure 3 shows the individual level results for each metric and task. This illustrates the individual variability that is observed in the responses to each of the stimuli. The largest individual differences are observed in the PRV LF/HF ratio.

The mean pulse amplitude was 36% lower (error: 28.8%) during the stress tasks (0.481) compared to the rest tasks (0.752). Based on a *t-test* this difference was significant ($\textit{p} < 0.04$). The mean vasomotion was 50% lower (error: 26.0%) during the stress tasks (21.1) compared to the rest tasks (42.6). Based on a *t-test* this difference was also significant ($\textit{p}<0.05$). No significant differences in PRV LF, HR or LF/HF ratio between the two tasks was observed.

**Figure 4:**
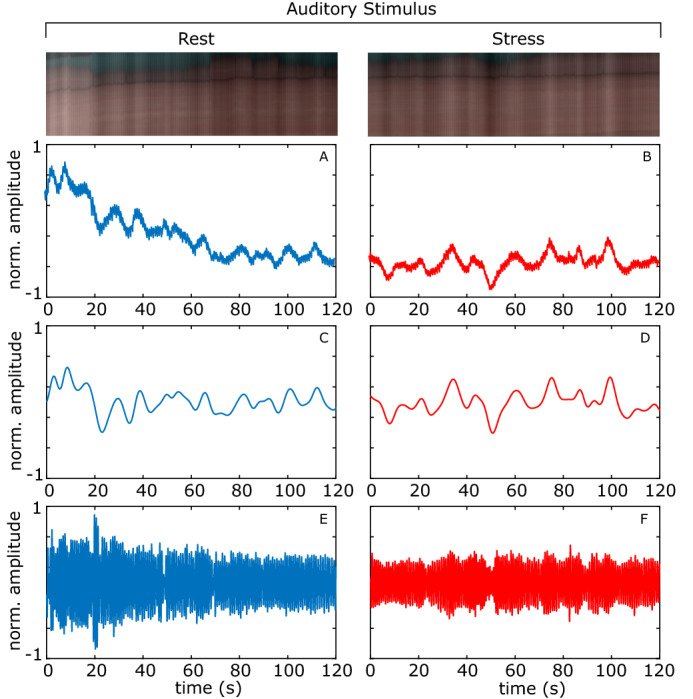
Auditory stimulation example. The total blood concentration ($\text {C}_{tb}$), vasomotion, and detrended peripheral pulse amplitude plots. A magnified scan line from the video is shown at the top. **A**, **B** Normalized total blood concentration at rest and under stress respectively. **C**, **D** Normalized vasomotion at rest and under stress respectively, **E**, **F** Normalized detrended peripheral pulse amplitude at rest and under stress respectively.

**Figure 5:**
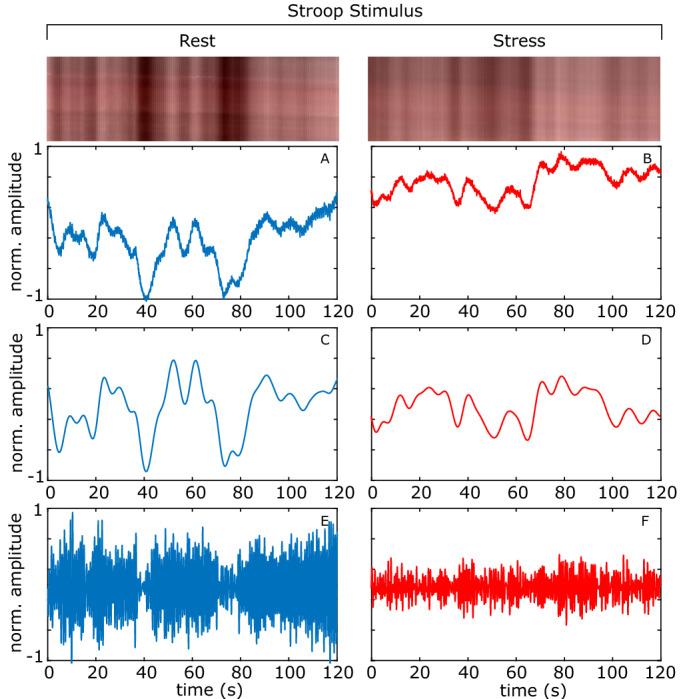
Stroop example. The total blood concentration ($\text {C}_{tb}$), vasomotion, and detrended peripheral pulse amplitude plots. A magnified scan line from the video is shown at the top. **A**, **B** Normalized total blood concentration at rest and under stress respectively, **C**, **D** Normalized vasomotion at rest and under stress respectively, **E**, **F** Normalized detrended peripheral pulse amplitude at rest and under stress respectively.

**Figure 6:**
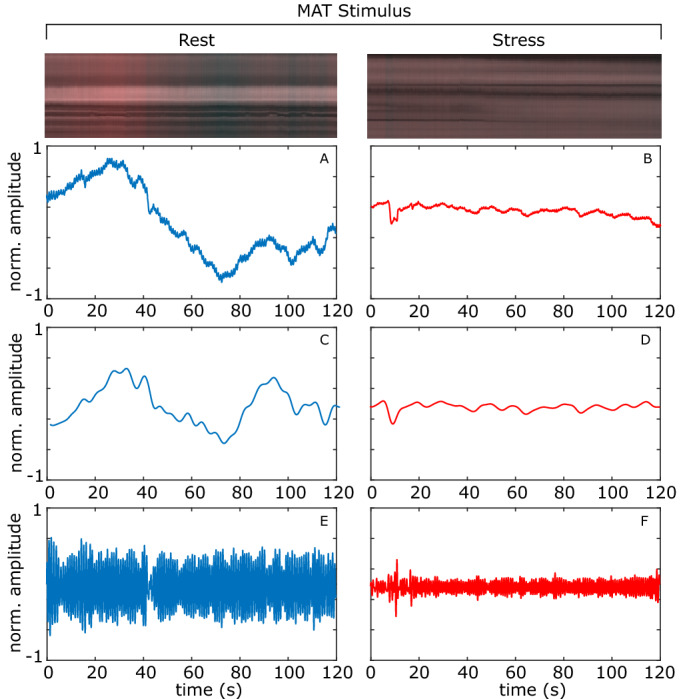
MAT example. The total blood concentration ($\text {C}_{tb}$), vasomotion, and detrended peripheral pulse amplitude plots. A magnified scan line from the video is shown at the top. **A**, **B** Normalized total blood concentration at rest and under stress respectively. **C**, **D** Normalized vasomotion at rest and under stress respectively. **E**, **F** Normalized detrended peripheral pulse amplitude at rest and under stress respectively.

Figures 4, 5, 6 shows the plots of the total blood concentration ($\text {C}_{tb}$), peripheral pulse amplitude and vasomotion for periods at rest and during stress for the auditory, stroop and mental arithmetic tasks respectively. Notice how the vasomotion shows less variability and the pulse amplitude decreases during the tasks. Figures 4, 5, 6 also show scan lines for the same video. The color changes have been magnified using EVM^39^ (applying the same filtering parameters as in the calculation of the vasomotion signal power). The reduced variability in the intensity across the scanline provides a qualitative example of how the light reflectance changes are less dramatic when the subject is under stress.

## Discussion

While electrocardiograms are the defacto gold-standard for measuring cardiovascular performance, peripheral hemodynamics provide complementary information. Autonomic nervous system activity has several different effects on the signals that can be measured peripherally. Our results show that the characteristics of peripheral pulse amplitude and low-frequency oscillations change during cognitive and psychological stress versus rest.

The changes in pulse rate variability low frequency, high frequency and LF/HF ratio were not significant across all experiments. There was high variability across participants with some showing higher LF/HF ratio in the stress condition and some showing higher LF/HF ratio in the rest condition. While increases in LF/HF ratio have gained acceptance as a tool used to capture changes in “sympathetic dominance” recent work has shown that this is based on assumptions which can be proven to be false^43^.

Our results suggest that peripheral pulse amplitude and vasomotion signals present a more reliable signal of sympathetic activation than PRV. Using imaging methods to measure pulse rate variability remains challenging. The noise in the measurement signals may have contributed to the lack of significance. This remains a reason why peripheral pulse amplitude and vasomotion measures may be more reliable for capturing changes in stress when using imaging methods. Measuring vasomotion changes is relatively easy using this approach, does not requires numerous processing steps and is not as sensitive to noise when compared to pulse rate variability. Furthermore, the peripheral pulse amplitude changes relatively quickly and could enable short-time window estimates. PRV and vasomotion are both signals that require longer time windows of at least 1 min as they focus on lower frequency signals.

There are many sources of variability in physiological states. While the trends across all experiments were similar and the overall effects significant, the three stimuli had somewhat different effects on the physiological signals. We were successfully able to measure the pulse signal, amplitude and vasomotion from both skin regions (hand and face). The mean vasomotion power and peripheral pulse amplitude were all greater in the pre-task condition compared to the task condition for both face and hands (see Table 1).

**Table 1:** Mean vasomotion power, peripheral pulse amplitude power, pulse rate variability low frequency (LF) power, high frequency (HF) power and LF/HF power ratio.

	Vasomotion		Pulse amp.		PRV LF/HF	
	Vol%$^2$		Vol%$^2$		No units	
	Rest	Task	Rest	Task	Rest	Task
Auditory	60.3 (42.2)	31.5 (14.0)	0.864 (0.509)	0.690 (0.225)	0.897 (0.397)	0.920 (0.520)
Stroop	48.6 (21.6)	29.0 (10.6)	1.130 (0.521)	0.563 (0.210)	0.458 (0.086)	0.374 (0.071)
MAT	20.5 (20.3)	3.71 (1.35)	0.272 (0.156)	0.208 (0.063)	0.370 (0.110)	0.513 (0.114)
All	42.6 (17.2)	21.1 (7.08)	0.752 (0.269)	0.481 (0.123)	0.565 (0.151)	0.593 (0.182)

The numbers in brackets reflect the standard errors.

The effects on vasomotion were most consistent for the auditory stimuli and least consistent for the MAT stimuli. This may be due to the different analyses measuring the physiological signals from different regions of the body, with the measurements from the face more noisy than those from the hand. Or may be due to the less consistent effect of the stimulus on the physiological state of the subjects. The effects on peripheral pulse amplitude were most consistent for the Stroop task.

Our results reveal quite large individual differences in all three signals: PRV, peripheral pulse amplitude and vasomotion. While there were significant decreases in pulse amplitude and the variance of the vasomotion signal, a single threshold would not necessarily lead to a reliable prediction of the level psychological stress.

Our experiments used contrived stimuli and were collected under controlled conditions. While this enables us to study responses in a more systematic fashion, changes in peripheral hemodynamics may be more subtle under real-world conditions and measurement is likely to be most challenging as lighting and body motions may not be carefully controlled. The cameras used in our experiment were higher-end digital cameras, while it is likely that the results would generalize to low-cost webcams (as has been shown in previous iPPG analyses^44^) and that equivalent hardware costs will drop in future, we cannot be sure that the sensitivity of all low-cost cameras would allow for measurement of the signals.

## Conclusions

Imaging-based physiological measurement is a powerful tool for quantifying cardiovascular performance. Peripheral hemodynamics, measured via the blood volume pulse and vasomotion, reveal information about autonomic nervous system activity. In order to investigate the effect of cognitive and psychological stress on peripheral hemodynamics we used a non-contact imaging photoplethysmography technique to measure blood flow during stressful tasks. We used a method, based on a tissue-like model of the skin, to extract hemoglobin $\text {C}_{HbO}$ concentration from videos of the skin during auditory, Stroop and mental arithmetic stimuli. From this we computed the pulse rate variability, peripheral pulse amplitude and vasomotion.

Our results show significant changes in peripheral pulse amplitude and vasomotion occur during periods of psychological stress. Pulse amplitude and the variability of vasomotion decreased during the stressful tasks. This supports previous findings but it is the first systematic exploration of peripheral hemodynamics using a non-contact, camera-based approach. While researchers have focused a lot on non-contact imaging photoplethysmography, the study of vasomotion and the effect of autonomic nervous system activity on this signal has received less attention. Our results suggest that vasomotion and peripheral pulse amplitude might be more robust measures of autonomic nervous system changes.

## Methods

### Principal behind a tissue-based model

**Figure 7:**
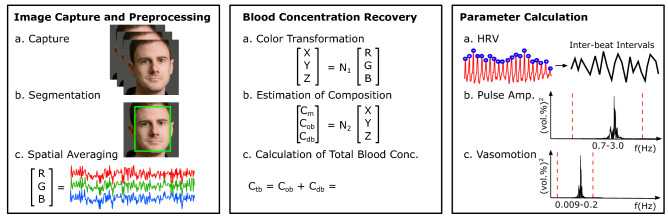
To evaluate peripheral hemodynamics we calculate pulse rate variability, pulse amplitude and vasomotion measures. We capture videos from digital cameras, segment a skin region (either on the face or on the hand) and calculate spatial averages of the color channel signals within the region of interest. We then recover the total blood concentration using a calibrated model-based approach. Finally we extract frequency domain measures of the physiological parameters. The face images used are examples provided with consent to be published in an online open-access publication.

We process each video according to steps in Fig. 7, based on a skin tissue model^23, 30^. We leverage this method directly as the performance of heart rate and heart rate variability measurement has been well validated^24^. Below we describe the method, given its importance for our subsequent analyses. Let us assume that skin tissue mainly consists of an epidermis (containing melanin) and a dermis (containing oxygenated and deoxygenated hemoglobin), the diffuse reflectance of skin tissue can then be expressed as:

1 $$\begin{aligned} I = S/S_{0} \end{aligned}$$

where, $\textit{S}_{0}$ and *S* are incident and detected light intensities, respectively. To express this more completely:

2 $$\begin{aligned} I = \left[ \int _{0}^{\infty } p_e(\mu _{s,e},g_e,l_e)\exp {(-\mu _{a,M}l_e)}dl_e) \right] \times \left[ \int _{0}^{\infty }p_d(\mu _{s,d},g_d,l_d)\exp {(-(\mu _{a,HbO} + \mu _{a,HbR})l_d)}dl_d)\right] \end{aligned}$$

The components: $\mu _{s}$, $\mu _{a}$, g, and l are the scattering coefficient, absorption coefficient, anisotropy factor, and photon path length, respectively. Then $p(\mu _{s}, g, l)$ is the path length probability function that depends on scattering properties and the geometry of the measurements.

### Color calibration

The differences in camera light sensitivity profiles and ambient lighting conditions mean that the RGB values measured by the camera need to be calibrated. The RGB-values in each pixel of the image are transformed into XYZ-values via a matrix $\mathbf{N} _1{:}$

3 $$\begin{aligned} \begin{bmatrix} X\\ Y \\ Z \\ \end{bmatrix} = \mathbf{N} _{1} \begin{bmatrix} \overline{R}\\ \overline{G} \\ \overline{B} \\ \end{bmatrix} \end{aligned}$$

This matrix $\mathbf{N} _\mathbf{1 }$ is determined via a Macbeth color checker standard (ColorChecker, X-Rite Incorporated, MI, USA) that has 24 reference color samples.

### Melanin and hemoglobin concentration estimation

Estimates of the concentration of melanin ($\text {C}_{m}$), oxygenated hemoglobin ($\text {C}_{ob}$) and deoxygenated hemoglobin ($\text {C}_{db}$) are made using the matrix $\mathbf{N} _{2}$. In determining the matrix $\mathbf{N} _{2}$, $p(\mu _{s}, g, l)$ and *l* for each layer are usually unknown. Therefore, we calculated 300 diffuse reflectance spectra *r*($\lambda$) in a wavelength range from 400 to 700 nm at intervals of 10 nm by an MCS for light transport in skin tissue under various values of $\text {C}_{M}$, $\text {C}_{HbO}$, and $\text {C}_{HbR}$ and, then, obtained the corresponding X, Y, and Z values. The absorption coefficient of melanin^45^ for $\text {C}_{M}$ was input to the epidermis as $\mu _{a,M}$. The sum of absorption coefficient for oxygenated hemoglobin, $\text {C}_{HbO}$, and that of deoxygenated hemoglobin, $\text {C}_{HbR}$ were $\mu _{a,H_{b}O}$ and $\mu _{a,H_{b}R}$. The layer thickness of epidermis and dermis were set to be 0.06 and 4.94 mm, respectively. The refractive index for each layer was assumed to be 1.4. These values have been used successfully in prior work^46^. The equations are then:

4 $$\begin{aligned} \begin{bmatrix} C_m\\ C_{ob} \\ C_{db} \\ \end{bmatrix} = \mathbf{N} _{2} \begin{bmatrix} \overline{X}\\ \overline{Y} \\ \overline{Z} \\ \end{bmatrix} \end{aligned}$$

where:

5 $$\begin{aligned} \begin{bmatrix} C_{m} \\ C_{ob} \\ C_{db} \\ \end{bmatrix} = \begin{bmatrix} \alpha _{0} &{} \alpha _{1} &{} \alpha _{2} &{} \alpha _{3} \\ \beta _{0} &{} \beta _{1} &{} \beta _{2} &{} \beta _{3} \\ \gamma _{0} &{} \gamma _{1} &{} \gamma _{2} &{} \gamma _{3} \\ \end{bmatrix} \begin{bmatrix} 1 \\ X \\ Y \\ Z \\ \end{bmatrix} \end{aligned}$$

The measurements of melanin and oxygenated hemoglobin and deoxygenated hemoglobin have been validated in prior work^23, 30^. For our analyses of changes in physiology with cognitive and psychological stressors we focus on the measurements of total blood concentration.

### Pulse rate variability

A 6th-order Butterworth filter is applied to the total $\text {C}_{tb}$ signal (cut-off frequencies of 0.7 and 2.5 Hz). A peak detection algorithm, see^18^, was used to detect the systolic peaks within the pulse wave. The resulting inter-beat intervals were filtered using a low pass filter with cut-off frequency 0.4 Hz. In this analysis we were interested in measuring the high (0.15–0.4Hz) and low frequency (0.04–0.15Hz) components of the PRV power spectra and therefore we filtered with a cut-off at 0.4 Hz. We construct the PRV spectrograms by calculating the Lomb-Scargle periodogram of the resulting in IBI time series. The LF/HF ratio is calculated as the ratio of power in the range 0.01–0.15 Hz and power in the range 0.15–0.4 Hz. The normalized powers (shown in Fig. 2) were calculated by dividing the LF and HF power by the total power in the range 0.04–0.4 Hz. The LF/HF ratio is dimensionless and so the normalization does not effect this measure.

### Plethysmogram/BVP

A 6th-order Butterworth filter is applied to the total $\text {C}_{tb}$ signal (low and high cut-off frequencies of 0.7 and 3 Hz, respectively). The resulting signal is the BVP estimate. We then compute the integral of the power spectrum for the signal within the same frequency band, to gain a measure of the amplitude of the signal.

### Vasomotion

A 6th-order Butterworth filter is applied to the total $\text {C}_{tb}$ signal (cut-off frequencies of 0.009 and 0.2 Hz). The resulting signal is the vasomotion estimate. We then compute the integral of the power spectrum for the signal within the same frequency band.

Three experiments were conducted each using different cognitive stressors to elicit autonomic responses from the participants in order to test the generalizability of the measurements to the stimulus. These stimuli have been previously validated to elicit psychological stress^47–49^. In all cases the participants reported experiencing higher stress during the stressors compared to the rest condition. We also analyzed two regions of interest the hand (experiments A and B) and the face (experiment C) in order to test generalizability of the measurements to the location on the body. All experiments were conducted indoors and with a mixture of sunlight and indoor illumination (Table 2).

**Table 2:** Summary of the three experiments in our study.

	Auditory stimulus	Stroop stimulus	MAT stimulus
Participants	10 (1 female)	11 (0 females)	11 (7 females)
Age ranges	22–43 years	22–30 years	18–30 years
Stimulus/task	Pure-tone sound at 1 kHz for 140 s	Stroop task with words at a rate of 1.5 s for 6 min	Subtracting 7 from 4000 repetitively for 2 min
Region of interest	Hand	Finger	Face
Camera	4-bit RGB CCD camera (DMK-21BF04, Imaging Source LLC, USA) with a camera lens (Pentax/Cosmica, Japan; f 16 mm, 1:1.4)	4-bit RGB CCD camera (DMK-21BF04, Imaging Source LLC, USA) with a camera lens (Pentax/Cosmica, Japan; f 16 mm, 1:1.4)	Olympus DSLR with a standard Zuiko 50 mm lens
Resolution/frame rate	640 × 480, 15 FP	640 × 480, 15 FPS	960 × 720, 30 FPS
Protocol	140-s rest period followed by 140-s task.	6-min rest period followed by 6-min task	120-s rest period followed by 120-s arithmetic task

We used three different stimuli to induce cognitive and psychological stress and recorded videos of the face or hand before and during these stimuli.

### Non-contact imaging validation

We used two datasets to validate the measurements the remote signal measurements. First, we used the dataset collected by Estepp et al.^50^. The dataset consists of 5 min recordings of 25 participants (17 males). Videos were recorded with a Basler Scout scA640-120gc GigE-standard, color camera, capturing 8-bit, 658 × 492 pixel images, 120 fps. The camera was equipped with 16 mm fixed focal length lens. Nine individuals were wearing glasses, eight had some form of facial hair (either beard or moustache), and four were wearing makeup on their face and/or neck. The participants exhibited the following estimated Fitzpatrick Sun-Reactivity Skin Types^51^: I-1, II-13, III-10, IV-2, V-0. Gold-standard physiological signals were measured using a BioSemi ActiveTwo research-grade biopotential acquisition unit. We used videos from a task that has motions most similar to those in the three datasets analyzed in this study, in which participants were seated still and exhibit small natural head motions.

**Figure 8:**
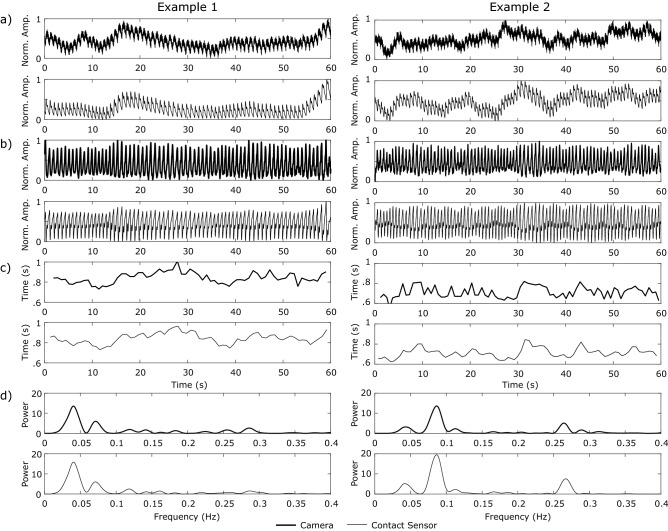
Physiological measurements using a camera (thick black lines) in comparison with a finger BVP contact sensor (thin gray lines). **a** PPG waveforms normalized from 0 to 1. **b** Detrended BVP waveform. **c** Inter-beat intervals (IBIs) formed by extracting the peaks from the BVP waveforms. **d** Normalized Lomb periodogram of the detrended IBIs. Notice in particular how the PPG waveform dynamics, amplitudes and inter-beat intervals are closely aligned.

We calculated the results on non-overlapping 1-min windows for the full set of 25 5-min recordings. The mean pulse waveform signal-to-noise ratio (calculated using the definition from de Haan and van Leest^52^) was 8.69 dB. The mean absolute error between the camera-based pulse waveform and the contact sensor pulse waveform was 0.11 (11%) and the mean error in heart rate estimation was 0.30 beats-per-minute (BPM). Examples of the raw pulse waveforms, the deterend pulse waveforms, the inter-beat intervals and the PRV spectra for two 1 min sections of the data are shown in Fig. 8. The Pearson correlation between PRV LF, HF and LF/HF ratio measured via the camera and the contact sensor was 0.85 ($\textit{p} < 0.01$), 0.87 ($\textit{p} < 0.01$) and 0.93 ($\textit{p} < 0.01$) respectively. Overall, these results show that it is possible to accurately capture the pulse signal from videos, especially in the case where there is little head motion and the ambient illumination is constant, as in all our experiments.

Second, we also compared the measurements captured during the MAT task in our own experimental dataset against contact sensor measurements captured simultaneously. For this dataset the Pearson correlation between PRV LF, HF and LF/HF ratio measured via the camera and the contact sensor was 0.93 ($\textit{p} < 0.01$), 0.93 ($\textit{p} < 0.01$) and 0.93 ($\textit{p} < 0.01$) respectively. Again, results validate that we can measure pulse signal accurately.

### Auditory stimulation

Our experiment featured ten healthy volunteers of both genders (1 female), different ages (22–43 years) and skin color. Informed consent was obtained from all participants prior to the data being collected. During the experiment participants were seated and the temperature of the room was held constant. For the stimulus each subject was exposed to a pure-tone sound at 1 kHz for 140 s. This protocol was approved by the institutional review board of Tokyo University of Agriculture and Technology and all methods were performed ion accordance with protocol.

#### Imager

A 24-bit RGB CCD camera (DMK-21BF04, Imaging Source LLC, USA) with a camera lens (Pentax/Cosmica, Japan; f 16 mm, 1:1.4) and standard white diffuser with 99% reflectance (SRS-99-020, Labsphere Incorporated, NH, USA) was used to acquire RGB color video at a resolution of $640 \times 480$ pixels. The diffuser was used to correct for the inter-instrument differences in the output of the camera and the spatial non-uniformity of the illumination. A ring-shaped polarizer and analyzer were used to reduce the specular reflection from the skin surface. The RGB images are acquired at a sampling (frame) rate of 30 Hz and recorded on a personal computer.

#### Pre-task

Participants were asked to sit still, and relax. The video and physiological recordings were captured for 140 s.

#### Task

Participants wore headphones and were exposed to a constant 1 kHz tone. The participants started the task immediately after the recordings were started. The video and physiological recordings were captured for 140 s.

### Stroop stimulation

Our experiment features 11 healthy male volunteers, different ages (22–30 years) and skin color. Informed consent was obtained from all participants prior to the data being collected. During the experiment participants were seated and the temperature of the room was held constant. This protocol was approved by the institutional review board of Tokyo University of Agriculture and Technology and all methods were performed ion accordance with protocol.

#### Imager

The same imaging set-up as in the auditory experiment was used.

#### Pre-task

Participants were asked to sit still, and relax. The video and physiological recordings were captured for 6 min (360 s).

#### Task

Participants were asked to perform the Stroop color-word test. The stroop Color-Word test is a task that causes cognitive conflict. The task has been well validated to induce mental stress. In the Stroop color-word test, color words in different colors are repeatedly shown on the monitor. Participants is told to say the name of the color of the word and not to answer with the color word itself. For example, if the word “RED” is shown in a green color, participant should answer “GREEN.” If participant answers “RED,” the system beeps. The interval between the words was 1.5 s. If the participant does not answer within 1.5 s the system also beeps. The video and physiological recordings were captured for 6 min (360 s). The participants started the task immediately after the recordings were started.

### Mental arithmetic stimulation

**Figure 9:**
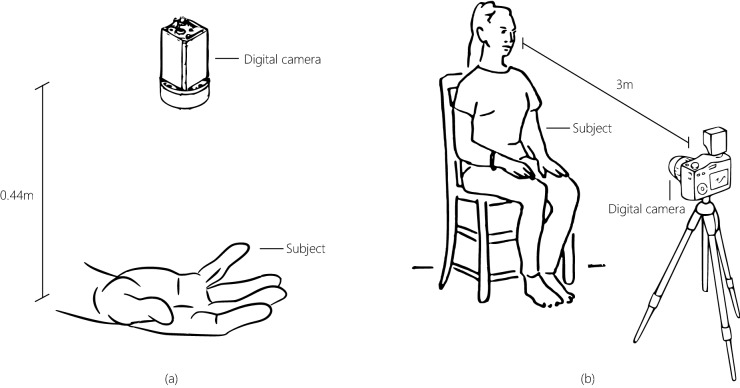
Experimental set-ups. Experimental set-up **a** was used for data collection in the auditory stimulation and stroop stimulation experiments. Experimental set-up **b** was used for data collection in the MAT stimulation experiment.

For this task participants were seated and the data were recorded on a laptop. Our experiments featured 11 participants (7 females) of different ages (18–30) and skin tones. Informed consent was obtained from all participants prior to the data being collected. The participants were facing the imager at a distance of approximately 3 m while the videos were recorded. The participant was seated and the temperature of the room was held constant. Figure 9 shows the set-up used to record the data. Recordings of the participants faces were taken for 2 min while contact measurements were simultaneously recorded (synchronously with the video frames). This protocol was approved by the institutional review board of Massachusetts Institute of Technology and all methods were performed ion accordance with protocol.

#### Imager

The camera used was composed of an Olympus DSLR body with a standard Zuiko 50mm lens. Videos were recorded at a sampling (frame) rate of 30 frames per second (fps) and a resolution of $960 \times 720$. The recording were in color (80-bit image with three channels $\times$ 16 bits/channel).

#### Pre-task

Participants were asked to sit still, look toward the camera and relax. The video and physiological recordings were captured for 2 min (120 s).

#### Task

Participants were asked to perform a mental arithmetic exercise silently for 2 min (120 s). Starting with the number 4,000 they were required to subtract seven sequentially as quickly and as accurately as they could. The video and physiological recordings were captured during this period. The participants started the task at the same time as the recordings were started. In order to increase the cognitive stress induced we told the participants that they were competing against other people to reach the lowest number. All the participants reported experiencing more stress during the task than during the pre-task period.
